# Prospective observational study investigating the effectiveness, safety, women’s experiences and quality of life at 3 months regarding cervical ripening methods for induction of labor at term—The MATUCOL study protocol

**DOI:** 10.1371/journal.pone.0262292

**Published:** 2022-01-21

**Authors:** Guillaume Ducarme, Stephanie Martin, Veronique Chesnoy, Lucie Planche, Marie-Pierre Berte, Elodie Netier-Herault

**Affiliations:** 1 Department of Obstetrics and Gynecology, Centre Hospitalier Departemental, 85000, La Roche sur Yon, France; 2 Clinical Research Center, Centre Hospitalier Departemental, 85000, La Roche sur Yon, France; Lausanne University Hospital: Centre Hospitalier Universitaire Vaudois (CH), SWITZERLAND

## Abstract

**Background:**

The purposes of successful induction of labor (IOL) are to shorten the time for IOL to delivery, increase the vaginal delivery rate, and reduce the rate of maternal and neonatal morbidity. In cases of unfavorable cervix (Bishop score <6), cervical ripening is advised to improve vaginal delivery rate. It may be initiated by mechanical (double balloon catheter (DBC), synthetic osmotic dilator) or pharmacologic (prostaglandins) methods, and the problem is complex due to the multitude of cervical ripening methods. We are constantly looking for the optimal protocol of cervical ripening for each woman. The present study aims to elucidate whether cervical ripening method is associated with increase rate of vaginal delivery, good women’s experience and unaltered long-term quality of life after cervical ripening at term regarding maternal and obstetric characteristics.

**Methods and design:**

The MATUCOL study is a monocentric, prospective, observational study of all consecutive women who required cervical ripening (Bishop score <6) using different methods (DBC, vaginal dinoprostone, oral misoprostol) with a live fetus at term (≥37 weeks) between January 2020 and August 2021. The outcomes will be mode of delivery, maternal and neonatal morbidity, discomfort/pain assessments during cervical ripening, women’s experience and satisfaction, and the impact of cervical ripening on the health-related quality of life at 3 months. If it reports a significant efficacy/safety/perinatal morbidity/women’s satisfaction/quality of life at 3 months post-delivery associated with a method of cervical ripening in a specific situation (gestational and/or fetal disease) using a multivariate analysis, its use should be reconsidered in clinical practice.

**Discussion:**

This study will reveal that some cervical ripening methods will be more effectiveness, safe, with good women’s experiences and QOL at 3 months compared to others regarding maternal and obstetric characteristics.

**Trial registration:**

This study is being performed at La Roche sur Yon Hospital following registration as GNEDS on January 8, 2020.

## Introduction

### Rational

In developed countries, 20–25% of pregnant women need induction of labor (IOL) [1]. The purposes of successful IOL are to shorten the time for IOL to delivery, increase the vaginal delivery rate, and reduce the rate of maternal and neonatal morbidity. Large, adequately powered, randomized controlled trials can be recommended to assess all these purposes. In cases of IOL with unfavorable cervix (Bishop score <6), cervical ripening is advised to improve vaginal delivery rate. Cervical ripening refers to the process of cervical modifications and uterine contractions that are initiated by mechanical (double balloon catheter, DBC, or synthetic osmotic dilator) or pharmacologic (prostaglandins) methods prior to the onset of spontaneous labor, and the problem is complex due to the multitude of cervical ripening methods used. The ideal method for cervical ripening has yet to be identified.

Naturally, each of the different mechanical or pharmacological induction method has a different mechanism of action, as well as safety and adverse-effect profile. Although data comparing different methods for IOL are available [2–5], there are insufficient comparative data to determine which of the methods is the most effective and safety profile with the best women’s experience and overall satisfaction at immediate and long-term. Variation in indications for IOL, parity, and gestational age at induction, as well as other demographic features, undoubtedly also affects clinical results. Variations in clinical practice, especially in case of consecutives methods, may also lead to an important delay between the onset of cervical ripening and the onset of the labor that can depreciate women’s well-being and satisfaction and induce morbidity. In order to try to tease out the nuances in safety and efficacy outcomes, large prospective observational studies are needed to assess whether methods is associated with more vaginal delivery, good women’s experience and good short- and long-term quality of life (QOL) at 3 months post-delivery in various maternal (obesity, previous diabetes, previous cesareans scar.) and obstetric (breech presentation, twins…) conditions, and for different indication for IOL (gestational diabetes mellitus, prolonged pregnancy, prelabor rupture of the membranes, pregnancy-associated hypertensive disorders, fetal growth restriction…). We are constantly looking for the optimal protocol of IOL for each woman before the widespread use of a specific device for a specific maternal or obstetric condition or IOL indication.

The present study aims to elucidate whether cervical ripening method is associated with increase rate of vaginal delivery, good women’s experience and unaltered long-term quality of life after cervical ripening at term regarding maternal and obstetric characteristics.

## Materials and methods

**Fig 1:**
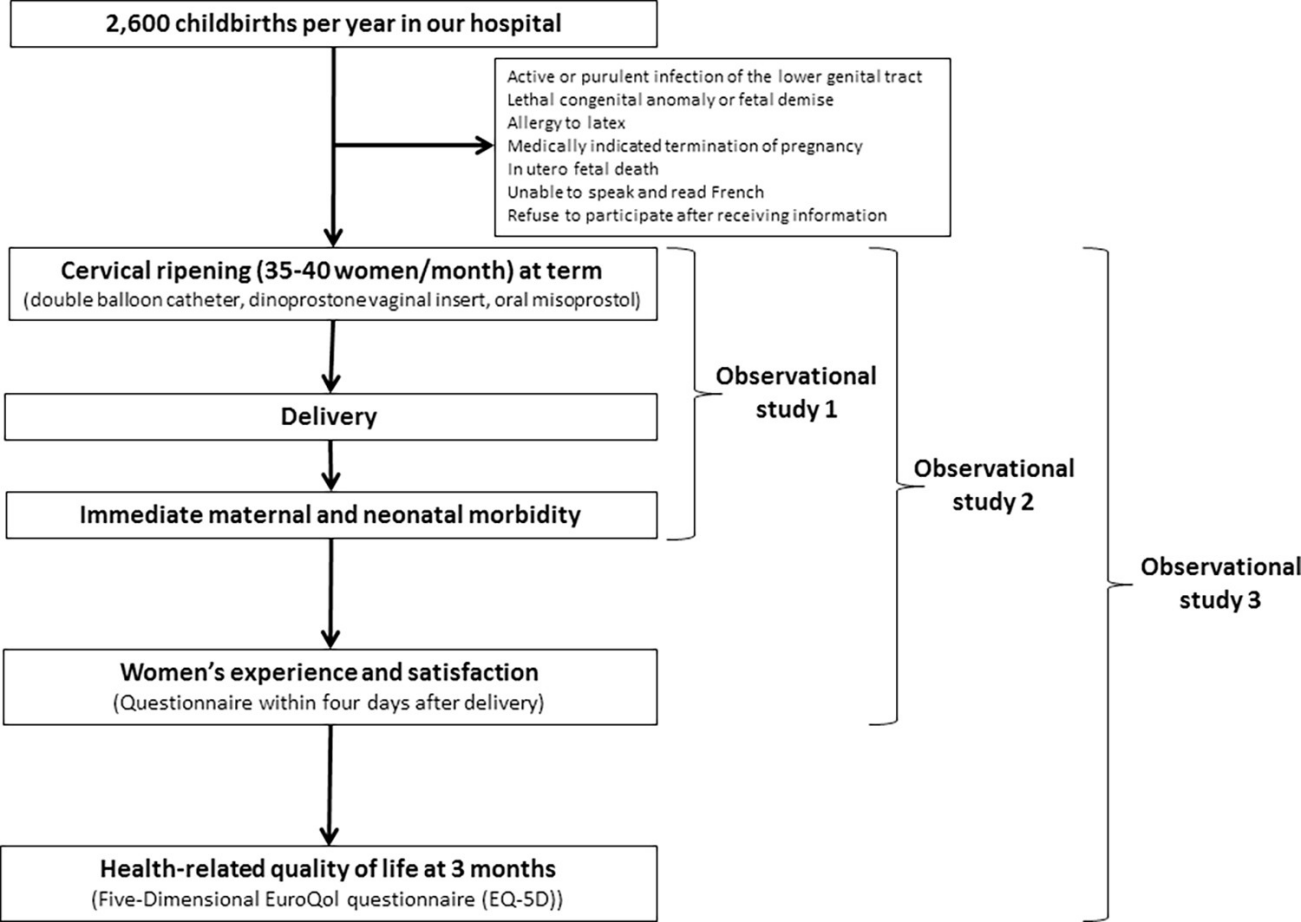
The study flowchart.

This is an ongoing, prospective, single-center, observational study that will run from January 9, 2020 to August 9, 2021. The study flowchart is presented in Fig 1. This study is being conducted according to the principals of the Declaration of Helsinki and the French approved guidelines. Information on the study will be provided to eligible women by obstetricians and midwives in labor ward immediately before cervical ripening. All participants received written information about the study using institutional review board-approved documents. Written consent is not required for prospective observational study according to the French law, but each eligible woman got the opportunity to decline the inclusion. After giving written information and oral consent, an anonymous number was allocated to each included women. The study protocol was reviewed and approved by a Research Ethics Committee *(Groupe Nantais d’Ethique dans le Domaine de la Santé* (GNED)) on 8^th^ January 2020 before the beginning of the study.

### Target population

All consecutive pregnant women who required device for cervical ripening before IOL with a live fetus, an unfavorable Bishop score (<6) and a normal pre-induction cardiotocograph (CTG) in a tertiary care hospital with more than 2,600 annual deliveries are consecutively included. The choice of the method of cervical ripening (DBC, vaginal dinoprostone insert or oral misoprostol) is left to the free discretion of the obstetrician responsible for the woman at the beginning of IOL.

#### Inclusion criteria

Women in the analysis must meet all inclusion criteria, as follows: over 18 years of age, placenta not closer than 2 cm from internal os, absence of undiagnosed vaginal bleeding, reassuring preripening CTG, cervical ripening for IOL whatever its indication, Bishop score <6, and gestational age ≥37 weeks at the time of intervention.

#### Exclusion criteria

Women exhibiting any of the following criteria are excluded: active or purulent infection of the lower genital tract, lethal congenital anomaly or fetal demise, allergy to latex, medically indicated termination of pregnancy, in utero fetal death, and unable to speak and read French.

### Trial design and follow-up

Participant registration for the study will run from January 9, 2020 to August 9, 2021, and women participation will run from January 9, 2020 to November 9, 2021. All women will require device for cervical ripening for IOL with an unfavorable cervix (Bishop score <6) with a live fetus at term whatever the indication for IOL. The choice of the cervical ripening method (DBC, vaginal dinoprostone insert or oral misoprostol) is left to the free discretion of the obstetrician responsible for the woman at the beginning of IOL, but in accordance with the institutional local guidelines for cervical ripening. DBC (Cervical Ripener Balloon®, Cook OB/GYN, Spencer, IN, USA) and vaginal dinoprostone insert (Propess®, Ferring, Saint-Prex, Switzerland) with 10 mg slow-release dinoprostone) are approved for use up to 24 hours. Oral misoprostol (Angusta®, Azanta, Valby, Denmark) may be used for more than 24 hours (20μg per day). If labor does not ensue or the Bishop score is still unfavorable (score<6) after 24 hours, there is no consensus and repeating the vaginal dinoprostone insert or oral misoprostol or switching to another method are options to the free discretion of the treating physician. The choice of the maximum duration of cervical ripening permitted before starting oxytocin if membranes are not accessible is also left to the free discretion of the treating physician with a maximum of 3 days in our obstetric team. Once Bishop’s score is ≥ 6, further management with oxytocin induction is recommended in our team.

Maternal sociodemographic characteristics, information regarding pregnancy follow-up, cervical ripening, standard perinatal outcomes, immediate women’s experience and satisfaction and long-term health-related QOL will be collected by one obstetrician (GD) from a prospectively maintained database of women who will be included throughout the study period.

Maternal characteristics collected will include maternal age, prepregnancy Body Mass Index (BMI, calculated as weight (kg)/[height (m)]^2^, based on height and the first weight noted in the obstetric record), women’s medical history (ie, obesity, previous bariatric surgery, previous diabetes, previous hypertension, one previous cesarean delivery…). Pregnancy and labor characteristics collected will include information regarding clinical characteristics at cervical ripening, including its indication, method and cervical status (Bishop score) before and after ripening, fetal presentation (cephalic or breech), uterine tachysystole, artificial rupture of membranes before starting oxytocin, labor (oxytocin induction, quantity of oxytocin used), delivery, and standard maternal and neonatal outcomes. Indications for cervical ripening will consist of prolonged pregnancy (over 41 weeks+4 days), prelabor rupture of the membranes, pregnancy-associated hypertensive disorders, fetal growth restriction (small-for-gestational-age, SGA), abnormality of fetal vitality (ie, oligoamnios or decreased fetal movements before 41 weeks of gestation), diabetes (gestational or previous; insulin‐ or non‐insulin‐dependent), macrosomia without diabetes, and other medical indication (ie, thrombocytopenia, intrahepatic cholestasis of pregnancy, hydramnios, other maternal complication). Pregnancy-associated hypertension disorders were determined by hypertension without proteinuria or preeclampsia (hypertension and proteinuria) after 20 weeks’ gestation in a previously normotensive woman [6]. GDM was diagnosed as usual, according to international guidelines for pregnant women [7], and women who will be not controlled with antenatal insulin therapy or who will present with an estimated fetal weight >97^th^ centile at 37 weeks were advised to undergo IOL at 39 weeks of gestation. Antenatal suspicion of SGA was defined as an ultrasonographic estimated fetal weight<10^th^ centile adjusting for gestational age and sex [8]. Antenatal suspicion of macrosomia was defined as an ultrasonographic estimated fetal weight>90^th^ centile adjusting for gestational age and sex [8]. Intrahepatic cholestasis of pregnancy is a cholestatic disorder characterized by pruritus with onset in the second or third trimester of pregnancy and elevated serum aminotransferases and bile acid levels.

Intrapartum variables collected will include gestational age at delivery, mode of delivery (spontaneous or operative vaginal delivery, caesarean section), the indication of cesarean delivery, the time of caesarean delivery (before labor (failure of IOL) or during labor (during the latent or active phase)), and birth weight. Maternal characteristics collected in immediate postpartum period will include severity of perineal tears, perineal hematoma, total estimated blood loss, postpartum hemorrhage (PPH, defined as bleeding 500 mL or greater) and severe PPH (defined as bleeding 1,500 mL or greater), need for additional uterotonic agent (sulprostone) and second-line therapies (Bakri balloon, uterine compression sutures, uterine artery embolization, and peripartum hysterectomy) for management of massive persistent PPH after failure of uterine massage and uterotonic agents to stop bleeding, chorioamnionitis, infections (defined by at least one of the following: endometritis, episiotomy infection, or wound infection requiring surgery), blood transfusion, thromboembolic event, intensive care unit (ICU) admission, and maternal death.

In addition, we will routinely measure newborns’ umbilical arterial blood gases at birth. Immediate neonatal morbidity data recorded will be 5-min Apgar score, cord pH, base excess, respiratory distress syndrome, neonatal jaundice, neonatal hypoglycemia, neonatal trauma (defined by the existence of at least one of the following criteria: fracture of the clavicle or a long bone, brachial plexus injury, and cephalhematoma), intraventricular hemorrhage, need for resuscitation or intubation, any transfer to the neonatal intensive care unit (NICU) for close monitoring of the neonate, sepsis, seizures, and neonatal death. Respiratory distress syndrome is defined by the presence of respiratory distress, as indicated by an increased oxygen requirement (FiO2 ≥0.4) and compatible chest radiographic findings without any evidence of another cause of respiratory distress. Neonatal hypoglycemia is defined as blood glucose <40 mg/dL in the first 24 h post-delivery or blood glucose <50 mg/dL from the second day of life. Neonatal hyperbilirubinemia will be recorded when the infant is treated with phototherapy after birth or admission at the neonatology department for this reason. Neonatal sepsis is defined as confirmed clinical infection with positive bacteriological tests (Observational study 1).

After birth, women’s experience and satisfaction will be evaluated with a self-questionnaire within four days after delivery. Included women are also informed that they will receive another questionnaire by mail at 3 months postpartum. The questionnaire in the immediate postpartum period consists of questions about women’s current feelings and experience of cervical ripening, labor and birth. First, among women with DBC or dinoprostone insert, they will be asked to indicate the discomfort/pain and experience during the insertion of the device, and while the system was in situ. The levels of discomfort/pain assessments will be rated on a visual analogue score 1–10. Second, we will ask all women to indicate the feelings about the duration of IOL. Women will be asked to answer this question on a 10-point Likert-like scale (“0 = too long and unbearable” to “10 = acceptable”). The specific experience about IOL and the women’s overall satisfaction regarding IOL, labor and delivery will be also explored with a 10-point Likert-like scale (“0 = very dissatisfied” to “10 = very satisfied”) (Observational study 2). The last question will also explore whether women would prefer the same cervical ripening method again in a future pregnancy. Breastfeeding when leaving the maternity will be also reported.

At 3 months postpartum, health-related QOL will be assessed based on women’s responses to the validated Five-Dimensional EuroQol questionnaire (EQ-5D) [9]. The EQ-5D is a generic, multi-attribute, preference-based measure. Its psychometric performance has previously been demonstrated in the maternity context [10]. The EQ-5D is a two-part instrument. The first is a descriptive system, which defines health-related QOL on the day of completion along 5 pre-specified dimensions: mobility, self-care, usual activity, pain or discomfort, and anxiety or depression. For each dimension, the respondent selects one of 5 levels of incapacitation: no, slight, moderate, severe, or extreme problems. The second part consists of a 20cm vertical visual analog scale (VAS) ranging from 100 (best imaginable health state) to 0 (worst imaginable health state), which provides an indication of the respondent’s own assessment of their health status on the day of completion. Women will complete a paper version of the EQ-5D questionnaire that will be mailed to them by the principal investigator (GD) within 1 week of the date on which their child reach 3 months of age, supplied with a self-addressed envelope for the response. In case of non-response no reminder will be sent (Observational study 3).

The data management and statistical aspects will be handled centrally by the Clinical Research Center of the hospital. Quality control will be conducted according to the standard operating procedures of the sponsor concerning studies in the investigational centers which comply with the Declaration of Helsinki and Good Clinical Practices.

## Endpoints

The primary endpoint in this study is the effectiveness (vaginal delivery), the safety (maternal and neonatal morbidity), and the women’s experience and overall satisfaction in immediate postpartum period regarding cervical ripening methods, maternal and obstetric conditions. The secondary endpoint is the impact of cervical ripening on the health-related QOL at 3 months according to the methods.

## Safety

Since this study is observational, there are no direct risks associated with participation. All cervical ripening methods were used in accordance with the French marketing authorizations.

## Statistical consideration

### Sample size estimation

The maternity unit delivers approximately 2,600 infants a year. We consider that 20% of women will meet the criteria for inclusion in the maternity unit, thus around 40 per month. An inclusion period of 20 months should make it possible to recruit 800 women, if we assume a participation rate of at least 95% and a 40% drop-out rate at 3 months.

### Statistical analysis

Continuous data will be described by their means ± standard deviations and compared by t tests (or Mann-Whitney tests when appropriate), and categorical data will be described by percentages and compared by χ^2^ tests (or Fisher’s exact tests when appropriate). Maternal and perinatal outcomes will be compared according to cervical ripening method. For the analysis of dissatisfaction in immediate postpartum period, a score < 5 will be considered as a negative result and defined as “dissatisfied”. Maternal QOL at 3 months, assessed by use of EQ-5D-5L scores estimated with the most up-to-date preference-based value set [11,12], will be summarized in each group using mean ± standard deviations and compared between groups using mean differences (99% confidence index). In the five domains of QOL, we will dichotomise into women reporting problems versus reporting no problems and will calculate the outcomes for the different groups. The associations between women’s dissatisfaction at immediate postpartum period and poor QOL at 3 months post-delivery and the characteristics of their pregnancy, cervical ripening methods, labor, and delivery will be then assessed with a multivariate logistic regression model. All analyses were carried out using R software (version 4.0.2). P values <0.05 were considered to be statistically significant.

## Discussion

This prospective observational study will include all women who will require cervical ripening whatever the method and the indication for IOL. It will assess data about the efficacy, safety, women’s experience of cervical ripening methods and health-related QOL at three months in different maternal and obstetric conditions and in different indications for IOL. The study will then identify factors associated with the efficacy (= vaginal delivery) of cervical ripening, safety, women’s experience and health-related QOL at three months. But, this database will also be used to analyze the efficacy, safety, and women’s experience of methods for cervical ripening in particular indication (gestational or fetal indication) or in particular women (previous cesarean delivery or obese)). We will expect to assess the “ideal” cervical ripening method in specific maternal and/or obstetric conditions. At long term, we also expect a better overall quality of life for the women after cervical ripening due to a personalized strategy.

The woman’s childbirth experience differs according to the type of labor (spontaneous or induced), the mode of delivery (vaginal route, cesarean section) and regarding parity [13]. In case of IOL, it is associated with less satisfaction compared to spontaneous labor [14]. Regarding woman’s experience during IOL, cervical ripening methods were questioned too [15–18]. In a secondary analysis of a randomized controlled trial that enrolled women undergoing IOL with oral misoprostol (n = 273, 54%) or Foley catheter (n = 229, 46%), experience of the duration of labor, pain during labor, general satisfaction with labor, and feelings of control and fear related to their expectation among term women were comparable between both groups. However, women in the Foley catheter group prefer more often to choose a different method for future inductions [18]. A French prospective cohort study including 94 hospitals and 3042 women who had an IOL showed that the determinants of maternal dissatisfaction were unbearable vaginal discomfort, insufficient pain relief during labor, lack of attention to the woman’s demands, a cesarean delivery and severe maternal complications [19]. These findings indicate the importance of advancing understanding of women’s perceptions of their childbirth experience during the perinatal period, specifically in case of IOL.

The woman’s childbirth experience may impact the future health of women and their children. A positive experience may lead to a sense of accomplishment and to a sense of dignity and self-confidence for the woman. A negative childbirth experience may rise to maternal distress, helplessness, even postpartum depression or post-traumatic stress disorder [20–23]. In addition, the woman’s childbirth experience also affects their early interactions with the child [24,25]. To our knowledge, no published study has evaluated the women’s satisfaction in the immediate postpartum period and the quality of life at long-term regarding various cervical ripening methods, maternal and obstetric conditions and various indications for IOL. The present study will seek to answer these important questions.

Strengths of our prospective observational study include its examination of cervical ripening for variety of maternal, fetal and elective indications at one tertiary hospital. All women with cervical ripening in our center will be cared for by the same obstetric team throughout the period of study, which especially avoided significant variation regarding diagnosis of maternal or obstetric diseases, parameters for cervical examination and IOL management, and obstetric intervention. This will allow for standardized protocols to be followed, thus mitigating differences in outcomes due to variations in clinical practice, increasing the generalizability of our findings. For example, cervical ripening is regularly used in women with one previous cesarean delivery in France [26–28]. In our team, women with one previous cesarean delivery are not contraindicated to mechanical device and its practice is usual and well framed [29]. Other strengths also include performance of individual and prospective collection of data throughout the study period by the same physician and using a standardized protocol according to Bishop score (systematic cervical ripening for IOL with an unfavorable cervix (Bishop score <6), repeating cervical ripening until Bishop score will be above 6). These methodological choices (monocentric design, very large inclusion criteria, outcome, sample size, questionnaires…) are guided by a pragmatic view about cervical ripening, such as all large prospective observational studies in obstetrics [30–33]. These studies allow multiple analyses of data and conclusions about pragmatic management and consequences of some current obstetric conditions that were not adapted to randomized controlled trials (i.e., twins with The JUmeaux MODe d’Accouchement (JUMODA) study) [30,34–39].

Nevertheless, the study presents certain limitations. First, the choice of the method of cervical ripening (DBC, vaginal dinoprostone insert or oral misoprostol) will be left to the free discretion of the obstetrician responsible for the woman at the beginning of IOL. That can introduce selection bias if participants regarding cervical ripening methods differ in important ways other than outcome. Second, our research will be not blinded, it is not possible to blind women and medical staff due to the nature of the intervention (e.g., CRB vs. vaginal dinoprostone insert vs. oral misoprostol), that may affect the physician’s strategy when choosing a follow-up plan. Third, the single-center design of the study will only allow us to conclude on our practice, and it will be difficult to extrapolate our results except among teams with the same practices as us. Fourth, it might also be interesting to analyze a potential economic impact with the consumption of several medication, analgesics and an increase in the hospital staying, specifically in case of consecutives methods. These secondary analyses were not originally planned, but should be done using registered data.

## Conclusions

This study will reveal that some cervical ripening methods will be more effectiveness, safe, with good women’s experiences and QOL at 3 months compared to others regarding maternal and obstetric characteristics.
